# A Study on Traditional Teaching Method Transferring to E-Learning Under the Covid-19 Pandemic: From Chinese Students' Perspectives

**DOI:** 10.3389/fpsyg.2021.632787

**Published:** 2021-03-11

**Authors:** Yuan Qing Jin, Chien-Liang Lin, Qun Zhao, Sung-Wen Yu, Yu-Sheng Su

**Affiliations:** ^1^College of Science and Technology Ningbo University, Ningbo, China; ^2^Department of Marketing and Distribution Management, National Pingtung University, Pingtung, Taiwan; ^3^Department of Computer Science and Engineering, National Taiwan Ocean University, Keelung City, Taiwan

**Keywords:** COVID-19, push pull mooring, online learning, e-learning, task technology fit

## Abstract

In response to the Covid-19 pandemic, online learning has been carried out in many countries with different types of online learning models being promoted and implemented. In the global pandemic continues, the education environment is forced to change from traditional classroom or blended teaching mode to online learning teaching model. With the outbreak of COVID-19, China was the first to announce that online courses are to be implemented in February 2020. In China, whether online learning can replace traditional offline teaching has become a topic worth discussing. Therefore, this study investigates university students in China by questionnaires and discussions of this topic. The study is based on the Push–Pull Mooring model. Based on 854 valid responses collected from an online survey questionnaire, structural equation modeling was employed to examine the research model. The results show that push effects (Perceived security risk, Learning convenience, and Service quality), pull effects (Usefulness, Ease of use, Teacher's Teaching Attitude, Task-technology Fit), and mooring effects (habit) all significantly influence users' switching intentions from offline to online learning platform. Finally, this study explores whether push–pull–mooring can be a reference for promoting and implementing online learning courses in Chinese colleges and universities in the future after the pandemic.

## Introduction

A Stable Development of Online Learning in recent years (Mulder and Janssen, 2013) has also prompted universities and teachers to use a variety of online learning techniques, such as Learning Management Systems, Internet-based technology for learning, Information, Communication and Technology (ICT), and Social Network-based Learning or mobile learning (Liao et al., 2019; Eksail and Afari, 2020; Huang et al., 2020), to help students learn by themselves and develop problem-solving skills then improve the effectiveness of traditional classroom teaching (Liu et al., 2010; Tian et al., 2014).

However, since early 2020, the new coronavirus has changed the mode of physical teaching or blended learning. The Ministry of Education of China also requires schools of all levels to respond to changes in the pandemic and adjust the form of classes. All universities are fully encouraged to use online teaching models (Cheng, 2020). Different from the traditional online teaching mode, which is a single course for online teaching, E-learning under pandemic is a learning model of emergency management. The online teaching course model has also changed from a single course to almost all the courses. Previous studies pointed out that during the COVID-19, the use of online learning has increased significantly, but the real effectiveness, and completion rate has not been significantly improved (Liu et al., 2020; Yang et al., 2020). This also shows the potential problem that online learning during the pandemic has an insufficient implementation. When the impact of the pandemic forced students to devote time and energy to familiarize the online teaching platforms, how to increase students' willingness to switch to online learning and reduce learning shocks is a key factor to think about during the pandemic. In previous studies, Chen and Keng (2019) investigated the Switching Intention of switching from traditional live-action English learning to online live-action English learning platform and suggested that online learning should pay more attention to the needs of learners in order to enhance the value of students' overall learning. However, unlike previous studies of Chen and Keng (2019), the willingness of students to switch from physical courses to online learning under the impact of the pandemic has also become an important topic to explain migration behavior under emergency management.

Therefore, there are two main reasons that the Push–Pull–Mooring (PPM) Model is used in this study to explore the Switching Behavior of Chinese college students using the online learning system. First, unlike traditional online learning environments, the changing from physical courses to online learning during the pandemic is also changing under the emergency, which is different from the traditional explanation that switching happens due to using habits. It also has theoretical value to explain migration behavior under emergency management. Second, previous studies explained students' online learning using behavior mainly applied theories such as Technology Acceptance Model (Huang and Teo, 2019; Ashrafi et al., 2020; Eksail and Afari, 2020), Expectation Confirmation Theory (Joo et al., 2018; Dai et al., 2020), and Extending The Unified Theory of Acceptance and Use of Technology (UTAUT2) (Tseng et al., 2019). However, different from the PPM theory, previous theories need to require fixed research frameworks and variables. PPM does not need fixed variables; the migration behavior in different environments is just conceptually explained in terms of Push, Pull, and Mooring.

By using PPM, we only need to consider the uniqueness of the research background and then determine the Push, Pull, and Mooring factors in different topics, which is more appropriate to exploring the transfer of Chinese college students from physical courses to online learning. Therefore, in response to this online learning Switching Behavior generated by emergency management, this study raises the following research questions: under emergency management, what are the key factors to Push, Pull, and Mooring of college students to transform the use from physical courses to online learning? Therefore, this study regards Chinese college students as the survey object and tries to find out factors that mainly promote the switching of Chinese college students.

## Literature Review

### Push–Pull–Mooring Model

The Migration of Population theory was originally conceived to understand the behavior of human migration (Lee, 1966; Moon, 1995). The timFmoe of migration can be short-term or long-term migration; by definition, it refers to people who work and live outside their homes for decades then choose to return to their place of origin at the end of their work. A long-term migration means that people leave their place of residence permanently and do not return (Jackson, 1986). According to the concept of migration theory, Lee (1966) proposed that the process of migration is affected by Push and Pull factors, and constructed the Push–Pull Model. The Push is considered to be influenced by external factors, such as lack of economic opportunity, lack of resources, and disease, which forces people to produce negative factors away from their place of origin, and Pull is the factors that drives people to move in, such as economic opportunity and political freedom. Then, Moon (1995) extended the Push–Pull Model, incorporated the Mooring effect, and put forward the Push–Pull–Mooring Model. The structure of the model indicated that people will be affected by Push, Pull, and Mooring factors during migration. Factors of Mooring are related to the migration process and may be factors that hinder or promote it. Mooring factors may have personal, social, or cultural values and are significant factors in migratory behavior. A summary of research topics on PPM theory in recent years is listed in Table 1. However, the framework of PPM must consider the unique characteristics of the research background to further identify factors of Push, Pull, and Mooring in different environmental contexts (Xu et al., 2014).

**Table 1:** Research topics on PPM theory.

**Authors**	**Research contexts**	**Push factor**	**Pull factor**	**Mooring factors**	**Dependent variable**	**Fundamental theories**
Hou et al. (2014)	Social networking service	Socializing, entertainment, system quality	Alternative attractiveness, peer influence	Switching costs, group cohesion	Switching intention	Pull–push–mooring
Hou and Shiau (2020)	Facebook to Instagram	Socializing, enjoyment, system quality, customer service satisfaction	Alternative attractiveness, peer influence, critical mass		Switching intention	Pull–push–mooring
Balakrishnan et al. (2017)	Social media for learning	E-learning perception	Social networking, convenience, social influence, academic reasons, ease of use	Barriers	Teaching and learning benefit	Pull–push–mooring
Li and Ku (2018)	E-commerce to social commerce	Low efficiency	Social presence, social support, social benefit, self-presentation	Conformity, personal experience	Switching intention	Pull–push–mooring
Xu et al. (2014)	Social networking service	Dissatisfaction with technical quality, dissatisfaction with information quality, dissatisfaction with entertainment value, dissatisfaction with socialization support, dissatisfaction with member policy	Attraction from the alternative SNS	Switching costs	Switching intention peer influence	Pull–push–mooring +expectation confirmation theory
Chang et al. (2014)	Social networking service	Regret, dissatisfaction	Alternative attractiveness	Switching costs	Switching intention	Pull–push–mooring
Zhang et al. (2012)	Blog	Overall dissatisfaction	Overall attractiveness	Sunk costs	Switching intention	Pull–push–mooring
Hsieh et al. (2012)	Blog to Facebook	Weak connection, writing anxiety	Enjoyment, relative usefulness, relative ease of use	Switching cost, past experience	Switching intention Actual behavior	Pull–push–mooring
Lai et al. (2012)	Mobile shopping	Inconvenience	Alternative attractiveness, peer influence	Low privacy and security, high switching cost, low trust	Switching intention Switching behavior	Pull–push-mooring
Jung et al. (2017)	Airline industry	Low service quality, pricing problem, low satisfaction, low trust	Attractiveness of alternatives, opportunity for alternatives, pricing benefits	High switching cost, low variety seeking, low prior switching experience, Involuntary choice	Switching intention	Pull–push–mooring

## Research Model and Hypotheses

### Research Model

**Figure 1:**
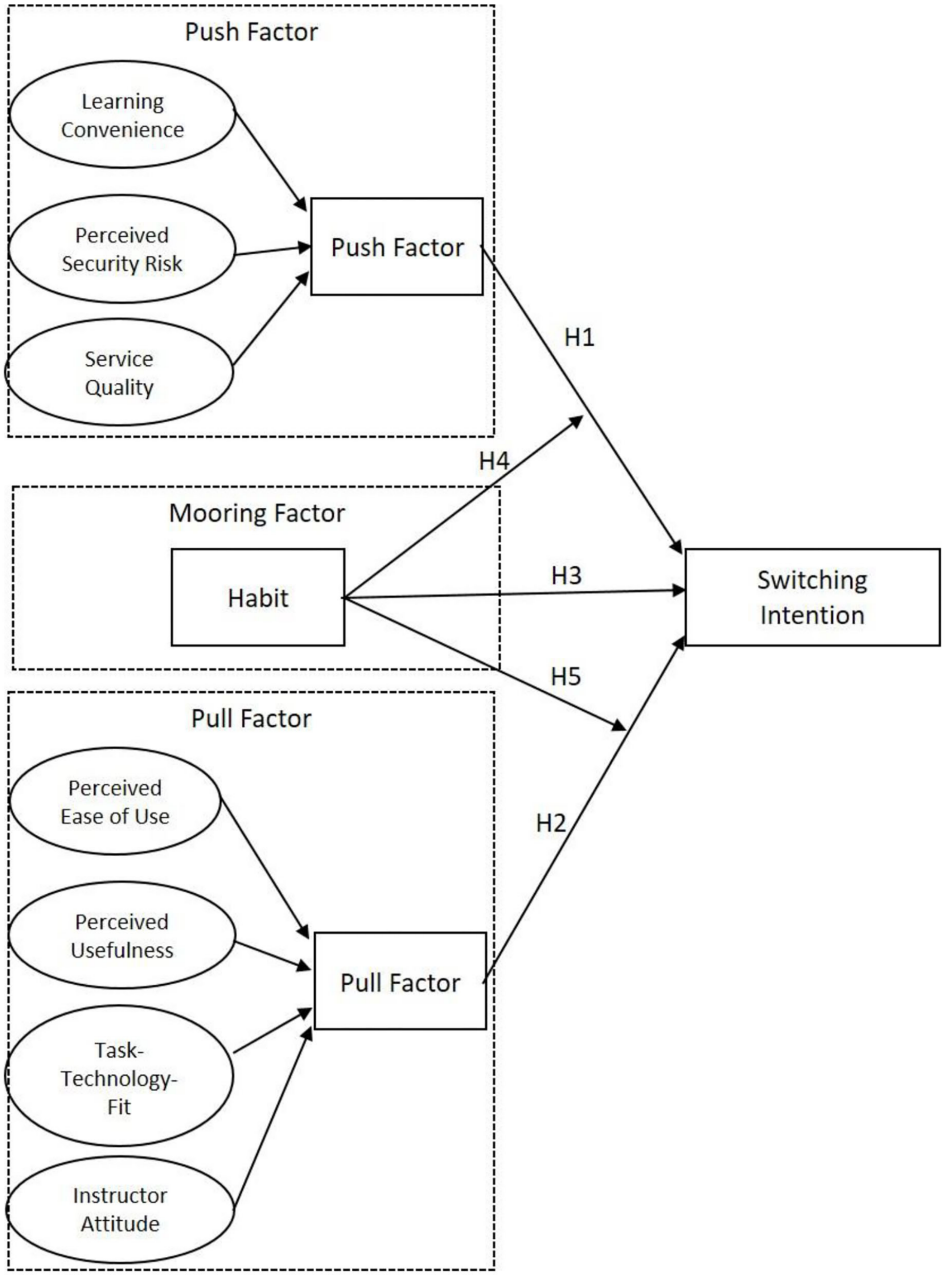
Research model.

Based on the literature of the previous PPM framework, this study attempts to construct a research model that explains the Switching Behavior of Chinese college students during the pandemic. This study defines the individual orientation of the Push, Pull, and Mooring factors according to the situation of the online learning environment. Figure 1 shows the main framework and assumptions of this study. The framework is presented as follows:

### Hypotheses

#### Push Factor

Convenience is defined as how service process is to save time, place, etc. (Brown, 1989). Compared to the traditional shopping process, online shopping offers consumers more convenience and saves more time and effort (Lai et al., 2012). With the development of information technology, the concept of convenience is to be able to learn online in unlimited space and time through various terminal devices and thus save time and cost (Carter and Campbell, 2012; Mangin et al., 2013; Hsu et al., 2018). However, the previous study also confirmed that convenience has an impact on the willingness to switch between physical and online learning environments (Michaelidou and Christodoulides, 2011). For online learning platforms, Chen and Keng (2019) believe that learners feel inconvenient for physical classes and that it has a negative impact on overall convenience. Thus, learners move the learning platform to the web and improve their own learning effect. For the same reason, considering the inconveniences of learning in the physical classroom, students will also be willing to switch to online learning because of the fear that they will be affected by the pandemic.

Security is the most direct factor that promotes and affects the trust of consumers in E-retailer (Grewal et al., 2003). Previous studies have found that risk will drive consumers to lower their willingness to use electronic services (Featherman and Pavlou, 2003; Nicolaou and McKnight, 2006). A high level of risk can also discourage users from using previous services (Yang and Lin, 2015). However, previous research has shown that when a new service has higher security, consumers would switch to that new service with higher security (Bhattacherjee and Park, 2014; Lai and Wang, 2015). For the definition of Security Risk, a previous study found that Security Risk refers to consumers' belief in potentially uncertain negative outcomes implied by online transactions (Kim et al., 2008). However, in PPM-related research, a previous study of Cheng et al. (2019) views Security Risk as a Push factor and defines Security Risk as “consumers exposed to a high level of Security Risk for previously used services,” and this risk “encourages consumers to switch to safer alternatives.” Therefore, this study believes that under the impact of the pandemic, Security Risk encourages students to reduce their chances of attending classes in physical classrooms and motivate them to increase willingness to join online learning.

For users of traditional information systems, the concept of Service Quality changed from traditional support for internal employees to support for external users, and service providers are paying more attention to providing more real-time, reliable, and valuable Focus on service. Service Quality is conceptualized as a service provider providing effective service to the person being served (Wang and Lin, 2012). Service Quality is a comparison between expectations and the actual performance, and good Service Quality provides a competitive advantage over competitors (Ladhari, 2009). Therefore, high Service Quality would positively affect customer value and satisfaction (Saeed et al., 2003; Cao et al., 2005; Lin, 2007) and then increase customer loyalty and willingness to purchase (Kim et al., 2004; Kuo et al., 2009; Wang and Lin, 2012). Surely, previous studies also regard Service Quality as an important factor affecting willingness of consumers to switch (Chang et al., 2017; Susanty et al., 2020; Tang and Chen, 2020). Although high Service Quality does not necessarily improve consumer loyalty, low Service Quality will keep customers away from old services (Jung et al., 2017; Liao et al., 2019). In the study of online learning switching behavior, the previous study of Chen and Keng (2019) used the PPM model to explore the learning Switching Behavior of physical English learning and indicated that service staff should effectively provide specific service of the English learning area to users. When users feel that the Service Quality of the English learning center is declining, they will also transfer to online English learning platform. Liao et al. (2019) also explore the willingness to switch learning to social networks, suggesting that learning platforms that fail to provide effective and quality services would also increase the willingness of learners to switch to other platforms. Therefore, for this study, during the pandemic the situation that students cannot return to the physical classroom results in the fact that physical classes could not provide real-time learning services to students, which promotes the willingness that students switch from physical study to online learning.

Based on the PPM framework, the Push factor is considered a negative influence factor that drives users to leave the existing services. Learning Convenience, Service Quality, and Safety Risks have proven to be push-oriented factors in previous studies (Chen and Keng, 2019; Cheng et al., 2019; Liao et al., 2019). According to the situation, this study regards three variables as the Push factors that affect the willingness of students to switch. Therefore, this study proposes the following hypothesis 1.

H1: The higher the learning convenience, service quality, and perceived security risk of offline learning services, the lower the likelihood that students will have learning switching intentions from offline to online.

#### Pull Factor

The concept of usefulness and ease of use originally derived from the scientific acceptance model (Technology Acceptance Model) proposed by Davis et al. (1989) and Davis (1989). It is considered that the user's intention to use is affected by perceived usefulness and ease of use. In the area of information system studies, most researchers demonstrated that cognitive usefulness and cognitive ease of use have positive effects on intention to use (Nysveen et al., 2005; Wakefield and Whitten, 2006; Castañeda et al., 2007; Wang and Lin, 2012). Also, Usefulness is defined as the concept that generally refer to an individual's use of an information system in order to improve the performance of his or her work. Ease of Use is defined as the ease of personal use of information systems (Davis, 1989; Davis et al., 1989). In addition, learners believe that using online learning systems can increase their learning performance, which urge learners more willingly to use online learning systems. Therefore, when learners think that using online learning systems is useful and easy to use, they are more likely to continue using online learning systems (Chang et al., 2017; Ayele and Birhanie, 2018; Huang and Teo, 2019; Huang et al., 2020). Therefore, this study regards the usefulness and ease of use about the services itself that are felt by Chinese college students using the online learning platform during the pandemic, which is the time when students are encouraged to be more willing to switch from physical classroom to online learning.

Goodhue and Thompson (1995) put forward the Task–Technology fit model to explain the success of information systems. Whether the performance of the information system meets the user's task requirements should be considered as the main factor to explain the job performance level. If the information system provides effective support, the usage would be increased and users' performance could be improved. By definition, technology is a tool or service that an individual uses to accomplish a particular task. From the perspective of information systems, technology refers to computer systems and services that support users' needs. Technology is a tool that could help users to finish specific tasks. Task, on the other hand, is viewed as the work to be accomplished by using technology (Goodhue and Thompson, 1995). Therefore, when the task meets the requirements, for users of information systems, task performance would be maximized (Khan et al., 2018). Looking back on studies about online learning, many researchers have also explored the relevance between the learners' performance and learning environments on the online learning platforms. The study found that task–technology fit would affect willingness to use and also learning performance (Yu and Yu, 2010; Lin and Wang, 2012; Kissi et al., 2018; Isaac et al., 2019; Zhang et al., 2019). Therefore, whether Tasks (learning goals under the pandemic) and Technology (online learning platforms) meet the learning needs of students will also affect students' switching to online learning.

The Instructor Attitude is defined as the learner's cognitive attitude from the teacher, including real-time response, teaching style, and teaching attitude that helps learners to learn through an online learning platform (Choi et al., 2007; Sun et al., 2008; Cheng, 2014). For the dimension of attitude, previous studies have confirmed that attitudes affect the willingness to adopt technology (Ayele and Birhanie, 2018; Huang and Teo, 2019; Huang et al., 2020). However, for the dimension of Instructor Attitude, studies of Sun et al. (2008), Al-Fraihat et al. (2020), and Rodríguez-Ardura and Meseguer-Artola (2016) indicate that teachers' teaching attitude is an important factor affecting students' learning satisfaction. Also, analyzing the environment of pandemic, whether teachers' teaching attitude under the pandemic can promote students' learning is also an important part of promoting students' switching to online learning.

Mainly in this study, the main factors that influence Pull factors include usefulness, ease of use, task–technology fit, and teachers' teaching attitude. These factors are all positive factors that provoke the willingness of switching. Therefore, this study proposes the following hypothesis 2:

H2: The higher the perceived ease of use, task-technology fit, instructor attitude, and perceived usefulness, the higher the likelihood the user will have offline to online learning platform-switching intentions.

#### Mooring Factor

Habit is an extremely important factor that affects consumer-switching intention. In particular, users are unlikely to meticulously compare and choose a relatively more advantageous service when they have become accustomed to using a specific service, and they tend to simply follow their current habits (Sun et al., 2017). Switching behavior-related studies noted that consumers generate inertia with existing services and prefer to maintain the “transaction relationship” of these services, to a certain extent, instead of actively looking for new services (Li, 2018; Chen and Keng, 2019). Research on information system-related applications also suggests that users remain unwilling to use new services despite their relative advantages, meaning that previous use habits have a negative impact on switching intention (Cheng et al., 2019). Therefore, previous use habits cause students to be prone to maintaining the status quo, which in turn contributes to a relatively low motivation to switch from offline to online learning. Accordingly, we propose the following Hypothesis 3:

H3: The higher the offline learning of habit, the lower the likelihood the students will have an offline to online learning platform-switching intention.

Among consumers, habit is a behavioral pattern produced by past habits (Li, 2018). Thus, regardless of the presence of an option, previous habits prompt consumers to stay with their current service providers, and consumers are often unwilling to change despite the availability of numerous other service plans (Kuo et al., 2013). However, mooring directly affects switching intention in the framework of PPM. In addition, previous studies have also indicated that mooring can weaken the relationship between push and pull factors in switching intention (Chen and Keng, 2019; Wang et al., 2019). Therefore, we assumed that previous use habits affect the relationship of the push and pull factors of students using online learning with their switching intention. Thus, we propose the following Hypotheses 4 and 5.

H4. The stronger the previous used habits, the weaker the relationship between the push factors and switching intentions.
H5. The stronger the previous used habits, the weaker the relationship between the pull factors and switching intentions.

### Construct Operationalization

A pilot test was conducted involving two associate professors, three Ph.D. Candidate, and 38 students to assess its questionnaire consistencies and ensure that the content of the question is face valid (Mokkink et al., 2010). Some professors and Ph.D. students were then invited to fill in, revise, and modify the first draft of the questionnaire so as to ensure the validity and applicability of this research. The content of the questionnaire was integrated with the content to ensure its validity. The operationalization of research constructs was implemented by using validated items from prior research. First, the scale was used for measuring second-order constructs of Push effects in offline to online learning, including Perceived Security Risk, Learning convenience, and Service Quality. Among them, Perceived Security Risk was measured by three items developed by Grewal et al. (2003); previous studies' Cronbach's alpha is 0.93. Learning convenience was measured by the three items of Chen and Keng (2019); previous studies' Cronbach's alpha is 0.93. Service Quality by the two items of Chen and Keng (2019) was measured; previous studies' Cronbach's alpha is 0.93.

Second, the scale was used for measuring second-order constructs of Pull effects in offline to online learning. Pull effects consist of four sub-constructs: Usefulness, Ease of use, Instructor attitude, and Task–technology Fit. The variables of perceived usefulness are assessed using a two-item scale, as suggested by Gefen et al. (2003); Cronbach's alpha is 0.89 in previous studies. Perceived ease of use is measured using a three-item scale, as adapted from Mohammadi (2015); Cronbach's alpha is 0.86 in previous studies. Teacher's Teaching Attitude was measured by three items developed by Rodríguez-Ardura and Meseguer-Artola (2016); previous studies' Cronbach's alpha is 0.85. Task–technology fit was measured by the four items of Isaac et al. (2019) fv; previous studies' Cronbach's alpha is 0.86. Habit was measured using items adapted from Chen and Keng (2019); previous studies' Cronbach's alpha is 0.80. Last, measures of switching intention were adapted from Chen and Keng (2019); previous studies' Cronbach's alpha is 0.95.

In addition, all items were measured along a seven-point Likert-type scale, ranging from 1 for “strongly disagree” to 7 for “strongly agree.” The items were first translated into Chinese by two information management professors and then translated back into English by another translator with special training in English-Chinese translation. Because the questionnaires were for distribution in China, the translation into Chinese allowed the respondents to read the items with no difficulty. One professional translator performed a back-translation to ensure that the original translation was content accurate.

### Data Collection

The formal survey was carried out after the pilot test. Users who have prior experience in switching from physical classroom learning to online learning and presently under the Covid-19 pandemic use online learning are valid respondents of this study. An online mode of data collection was selected because of its advantages in expediency in data collection, fast response time, cost efficiency, and the ability to reach a wide population of users (Bhattacherjee, 2002; Shiau and Luo, 2012).

The questionnaire of this study was posted on a survey website, wjx (https://www.wjx.cn/), which is a professional online survey service in China. The survey, conducted between May and June 2020, focused on Chinese universities that adopted full online learning during the COVID-19 pandemic. To ensure the validity of the questionnaires collected, we confirmed whether the students who answered the questionnaires had attended classes using online learning for 9 weeks or longer during the pandemic. Furthermore, to ensure the reliability of the questionnaires, we employed convenience sampling and snowball sampling and collected the samples by sharing the questionnaires with teachers with other universities and with our own university colleagues in WeChat groups, who then distributed the questionnaires to the students in their courses. The questionnaires were answered by the students voluntarily and then collected. To encourage participation and improve response, a nominal incentive of 3 RMB was offered to every respondent who provided complete answers.

Considering that the respondents did not understand the intention and perception of switching from offline to online courses, a simple question was designed and placed at the beginning of the questionnaire to help respondents review their switching behavior during the pandemic. A total of 870 questionnaires answered by the students of 18 universities in eight provinces were collected through wjx. Finally, after excluding the 16 invalid questionnaires, we collected 854 valid questionnaires to be used for data analysis.

Of all the respondents, 238 are male (27.87%) and 616 are female (72.13%). The majority of respondents are graded between the first and second year (79.28%). Before the Covid-19 pandemic, 57.49% report having never experienced an online learning experience, and the rest (42.51%) have had more than 6 months of experience. Additionally, nearly 46.25% of the respondents spend below 1 h of time spent per day on online learning, while 14.87% spend over 4 h. Finally, about the institutions of the students who filled in the questionnaire, 372 (43.56%) students belong to the management/business school, 114 (13.35%) from the humanities school, 77 (9.02%) from the law school, 82 (9.6%) from the science school, 96 (11.24%) from the engineering school, and 113 (13.23%) from the education school. For the type of colleges, 546 (63.93%) were from the government-funded colleges/universities and 308 (36.07%) from the government-funded colleges/universities.

## Results

SmartPLS 3.2.8 package was used to test the hypotheses in our research model (Ringle et al., 2015). This research model employs a second-order model with a reflective–formative type (Becker et al., 2012). In this study, push factors and pull factors were defined as a secondorder formative construct. The construct among Push factors included three reflective dimensions–Perceived security risk, Learning convenience, and Service quality. The construct for pull factors included four reflective dimensions–Perceived ease of use, Perceived usefulness, Task-technology Fit, and Instructor attitude. However, to test a second-order formative model, PLS-SEM is the appropriate choice because AMOS is not able to test a second-order formative model (Shiau and Chau, 2016; Huang et al., 2017; Hair et al., 2018, 2019; Shiau et al., 2019). Therefore, the PLS approach is appropriate for this research model analysis.

Common method variance is related to the measurement method and does not originate from the construct represented by the measurement item itself (Williams and Anderson, 1994; Williams and Brown, 1994; Podsakoff et al., 2003) and thus may cause measurement errors. Therefore, to reduce the problem of common method variance, two methods were applied. First, at the data collection stage, the questionnaire was deliberately processed through a paginated approach to provide respondents with an appropriate rest time between each page, thereby reducing the impact of common method variance caused by continuous same scale through time differences (Podsakoff et al., 2003). Second, Harman's single-factor test was used to verify whether there is a common method variance (Podsakoff et al., 2003). Principal component factor analysis was performed, and the results excluded the potential threat of common method variance. Because no single factor explained more than 50% of variance, this study did not exhibit a significant level of common method variance, and the results were within an acceptable range (Shiau and Luo, 2012).

Multicollinearity. According to the literature suggestion by Hair et al. (2017), value tolerance has a threshold of 0.10 and a VIF value below 5. Table 2 presents the all construct VIF values range from 1.85 to 2.99, which show that the results of this study meet the requirements.

**Table 2:** The analysis results of Factor loading, Cronbach alpha, composite reliability & AVE.

**Construct**	**Items**	**Factor loading**	**α**	**CR**	**AVE**	**VIF**
Perceived security risk (SER)	SER1	0.96^***^				
	SER2	0.95^***^	0.95	0.97	0.90	1.64
	SER3	0.94^***^				
Learning convenience (LC)	LC1	0.89^***^				
	LC2	0.93^***^	0.91	0.94	0.84	1.96
	LC3	0.94^***^				
Service quality (SQ)	SQ1	0.97^***^				
	SQ2	0.97^***^	0.94	0.97	0.94	1.70
Habit (HABIT)	H1	0.93^***^				
	H2	0.95^***^	0.92	0.95	0.86	1.94
	H3	0.91^***^				
Perceived ease of use (EOU)	EOU1	0.92^***^				
	EOU2	0.95^***^	0.94	0.96	0.89	2.57
	EOU3	0.96^***^				
Perceived usefulness (PU)	PU1	0.96^***^				
	PU2	0.97^***^	0.93	0.96	0.93	3.49
Task-technology Fit (TTF)	TTF1	0.94^***^	0.94	0.96	0.90	4.09
	TTF2	0.96^***^				
	TTF3	0.95^***^				
	TTF4	0.91^***^				
Instructor attitude (IAT)	IAT1	0.93^***^				
	IAT2	0.93^***^	0.92	0.95	0.86	2.54
	IAT3	0.94^***^				
Switching intention (SW)	SW1	0.92^***^				
	SW2	0.94^***^	0.94	0.96	0.85	DV
	SW3	0.90^***^				
	SW4	0.92^***^				

### Measurement Model

The measurement model is used to examine reliability, convergent validity, and discriminant validity (Liang and Shiau, 2018; Shiau et al., 2020). Internal consistency can be assured by examining the composite reliability of the constructs (Fornell and Larcker, 1981). As shown in Table 6, Cronbach's α value for all constructs was well above the recommended threshold of 0.70 (Hair et al., 2019) and ranged from 0.91 (LC) to 0.95 (SER). The composite reliability (CR) value was > 0.7 and ranged from 0.94 (LC) to 0.97 (SER) (Hair et al., 2017). The roh_A value for each construct was well above the recommended threshold of 0.7 (Dijkstra and Henseler, 2015), and obtained values ranged from 0.91 (LC) to 0.95 (SER). The results indicated that our measurement model had good internal consistency.

Convergent validity can be evaluated by checking whether the average variance extracted (AVE) values are larger than 0.5 (Fornell and Larcker, 1981) and ranged from 0.84 (LC) to 0.94 (SQ). All of the factor loadings of the all items are significant and > 0.7 (Hair et al., 2017). Therefore, the convergent validity of these measures is satisfied, as shown in the Table 2 below.

The discriminant validity of the constructs was evaluated using the approaches evaluated by Fornell and Larcker method (1981) and heterotrait–monotrait (HTMT). All square roots of the AVE values are higher than all the correlation coefficients shown, which again indicates the appropriate discriminant validity of these measures (Fornell and Larcker, 1981; Chin, 1998). Second, Henseler et al. (2014) proposed the heterotrait–monotrait (HTMT) ratio of the correlations. Henseler et al. (2014) suggested 0.90 as a threshold value for structural models with constructs. In this research, the values ranged from 0.43 to 0.837, which indicated that discriminate validity was established for all constructs of the model, as shown in Tables 3, 4. The results indicated that our measurement model had acceptable discriminant validity.

**Table 3:** Analysis of discriminant validity (Fornell–Larcker Criterion).

	**Habit**	**TTF**	**LC**	**SER**	**IAT**	**EOU**	**PU**	**SQ**	**SW**
Habit	**0.93**								
TTF	0.53	**0.95**							
LC	−0.61	−0.66	**0.92**						
SER	−0.44	−0.44	0.60	**0.95**					
IAT	0.62	0.73	−0.57	−0.29	**0.93**				
EOU	0.44	0.71	−0.60	−0.40	0.71	**0.94**			
PU	0.46	0.83	−0.65	−0.48	0.64	0.70	**0.97**		
SQ	−0.77	−0.63	0.62	0.51	−0.67	−0.53	−0.56	**0.97**	
SW	0.48	0.75	−0.66	−0.56	0.56	0.60	0.73	−0.58	**0.92**

*SER, Perceived security risk; LC, Learning convenience; SQ, Service quality; Habit; EOU, Perceived ease of use; PU, Perceived usefulness; TTF, Task-technology Fit; IAT, Instructor attitude; SW, Switching intention. Values in the diagonal (bold) are square root of the AVE*.

**Table 4:** Analysis of discriminant validity (Heterotrait–Monotrait).

	**Habit**	**TTF**	**LC**	**SER**	**IAT**	**EOU**	**PU**	**SQ**	**SW**
Habit									
TTF	0.57								
LC	0.66	0.71							
SER	0.46	0.47	0.64						
IAT	0.67	0.78	0.62	0.31					
EOU	0.47	0.75	0.65	0.43	0.76				
PU	0.50	0.87	0.71	0.52	0.69	0.75			
SQ	0.83	0.67	0.67	0.54	0.72	0.56	0.60		
SW	0.51	0.79	0.71	0.59	0.60	0.64	0.78	0.62	

*SER, Perceived security risk; LC, Learning convenience; SQ, Service quality; Habit; EOU, Perceived ease of use; PU, Perceived usefulness; TTF, Task-technology Fit; IAT, Instructor attitude; SW, Switching intention*.

In Table 5, the formative measures of second-order constructs were assessed based on the significance (*p* < 0.05) of their weights, which indicated their contributions to the corresponding second-order constructs (Wang and Haggerty, 2011). As shown in Table 5, the three dimensions of Push factors (Perceived security risk, Learning convenience, and Service quality) and the four dimensions of Pull factors (Perceived Usefulness, Perceived ease of use, Instructor attitude, Task-technology fit) significantly contributed to their constructs in this context. Thus, we found support for the dimensions of the push and Pull factors as theorized and tested within our sample.

**Table 5:** Result of weights.

**Construct**	**Sub-construct**	**Weights**
Push factors	Perceived security risk (SER)	0.438^***^
	Learning convenience (LC)	0.444^***^
	Service quality (SQ)	0.293^***^
Pull factors	Perceived ease of use (EOU)	0.265^***^
	Perceived usefulness (PU)	0.202^***^
	Task-technology Fit (TTF)	0.402^***^
	Instructor attitude (IAT)	0.247^***^

* *p < 0.01*.

### Structural Model

**Figure 2:**
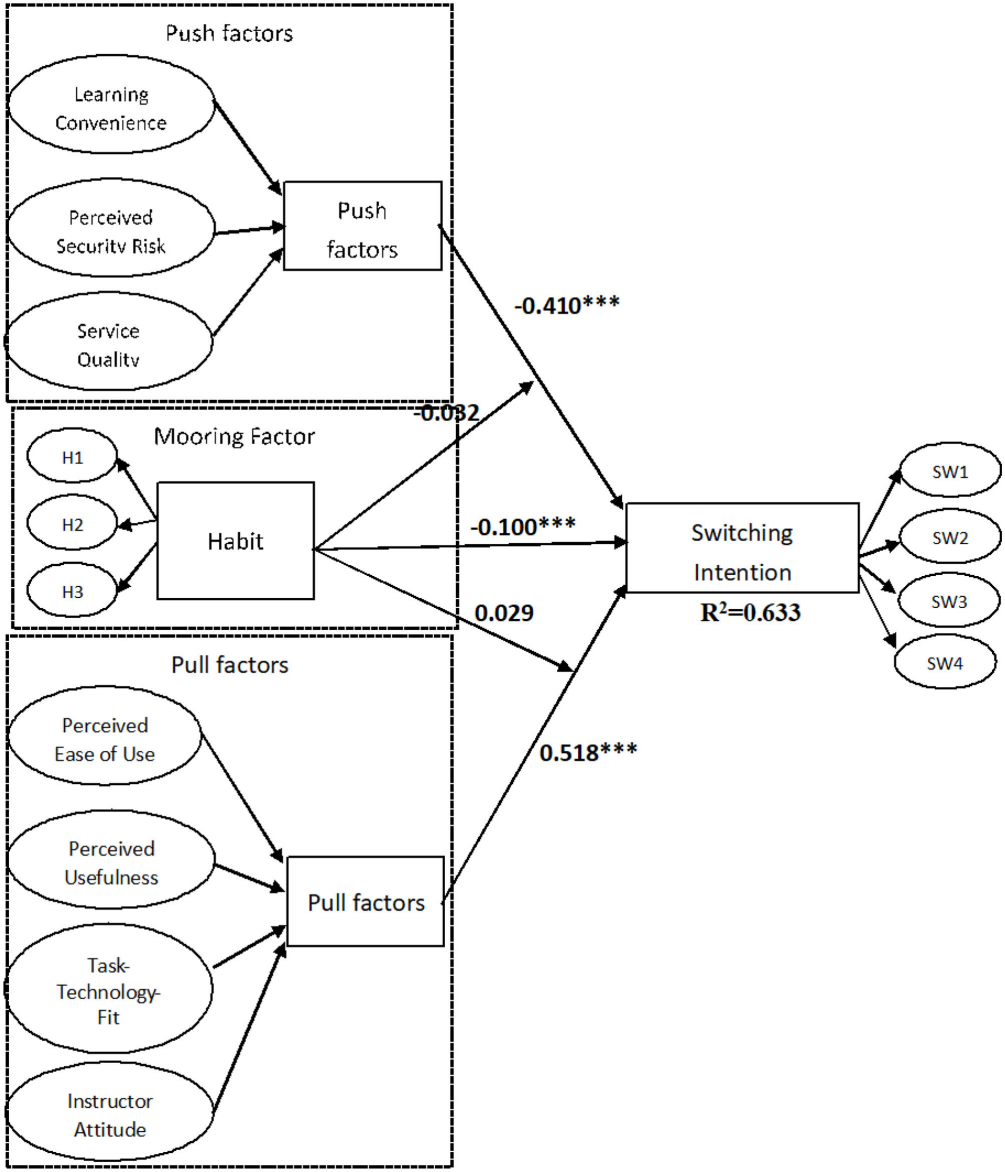
PLS results of the research model.

To test our hypotheses, a bootstrap technique resampling procedure was used to examine the stability of the PLS estimates, using resamples of 5,000 (Hair et al., 2017). Figure 2 shows the results. Overall, the research model explained 63% of the variance in Switching Intention to adopt online learning. These results show that the proposed research model has a rather high explanatory power and provides substantial support for H1 through H5.

The empirical results support hypotheses H1, H2, and H3. Push effect (H1) has a negative significant influence switching intention (β = −0.410, t = 8.55, *p* < 0.01). Second, the pull effect (β = 0.518, t = 11.25, *p* < 0.01) has a positive significant influence on switching intention, thu5s supporting H2. We also found that Habit (β = −0.100, t = 2.530, *p* < 0.05) has a strong negative influence continuance intention, as posited in H3. According to the results, H4 and H5 concern the moderating effects of habit on the relationship between the Push factors and Pull factors on switching intention. In addition, the results reveal that the moderating of the mooring effect on the push effect (β = −0.032, t = 1.632, *p* > 0.05) as well as that on the pull effect (β = 0.029, t = 1.75, *p* > 0.05) on switching intentions, has no significant influence. Therefore, this results not support H4 and H5 (see Figure 2).

In Table 6, according to Hair et al. (2017), effect size f2 is assessed as 0.02 representing small, 0.15 representing medium, and 0.35 representing large. Table 6 shows the effect size f2 of the endogenous constructs. Table 6 shows all f2 results according to effect sizes: one relationship (large), one (medium), one (small), and other variables have no relationship. Based on the blindfolding procedure, Stone–Geisser's Q^2^ value measures the predictive relevance of the model, where Q^2^ values of 0.02 represent small, 0.15 represent medium and 0.35 represent large (Hair et al., 2017). According to Hair et al. (2014), the Q^2^ values of all endogenous variables were over zero, suggesting that the model has predictive relevance for switching intention (Q^2^ = 0.55) (see Table 6).

**Table 6:** Effect size.

**Hypothesis**	**Effect size f^**2**^**	**Q^**2**^**
H1: Push factors → SW	0.17	0.55
H2: Pull factors → SW	0.35	
H3: Habit → SW	0.02	
H4: Habit*Push → SW	0.00	
H5: Habit*Pull → SW	0.00	

*SW, Switching intention*.

## Discussion and Conclusions

### Practical Implications

From the perspective of university management, an online learning environment under emergency management actually represents an advantageous turning point in promoting the development of online learning. Push factors consisted of perceived security risk, learning convenience, and service quality, and we believed that push factors were negative motivations for students to switch from offline to online learning. Thu5s, when students experienced the push factors formed by the safety, service quality, and convenience of offline learning during the pandemic, they chose not to attend the physical classrooms. This result was consistent with the switching intention observed in previous PPM studies (Chen and Keng, 2019; Cheng et al., 2019). Next, according to the results, because push factors were a second-order formative construct, learning convenience was discovered to be the major influencing factor that led to push factors, followed by perceived security risk and finally service quality, which was the least influential. The students chose to switch to an online learning environment, with learning convenience among the push factors playing a prominent role. This result was also consistent with previous results on the switch from offline learning to live online English learning (Chen and Keng, 2019). In particular, the inconvenience of transportation during the pandemic resulted in regional control in many cities and regions due to the pandemic and was a key factor prompting students to switch to online learning. Security factors were another major indicator producing push factors that caused students to leave offline courses (Cheng et al., 2019). In particular, due to the pandemic, students concern that their university's lack of security measures in arranging offline courses caused students to prefer to switch to online classes. Finally, service quality was an additional push factor, a result that was also consistent with that of previous studies that discussed the switch from offline to online learning (Chen and Keng, 2019). Therefore, we believed that when teachers cannot provide students with learning and support through physical classes due to the pandemic, students choose to switch to online learning platforms because their learning needs cannot be met. Overall, the results of push factors, which influenced student behavior in leaving the physical classes and switching to online learning, revealed that the inconvenience and inadequate service quality of physical classes as well as safety considerations during the pandemic were all essential push effects leading to online learning.

Pull factors are mainly a second-order formative construct composed of four sub-constructs: perceived ease of use, perceived usefulness, task–technology fit, and instructor attitude. Pull factors, the result of the positive effects of the shift to online learning, can be described as the attraction results of targetable attributes and characteristics. In this study, pull factors were based on perspectives of student learning effectiveness when they used online learning platforms. According to the results, pull was mainly affected by task–technology fit, a finding that is consistent with the results of previous studies on the use intention of online learning (Khan et al., 2018; Isaac et al., 2019). Student learning goals during the pandemic were to complete their learning tasks through online learning. Therefore, these learning tasks should be considered in switching intention (to online learning). The choice of platform was markedly important. Next, perceived ease of use and perceived usefulness also affected student switching intention to online learning during the pandemic, an observation that was consistent with the results of previous discussions on switching intention to online learning (Balakrishnan et al., 2017; Cheng et al., 2019). Accordingly, during the pandemic, the functionality and operability of online learning as well as the platform-delivered course content selected by teachers and universities were leading attributes that facilitated switching intention. In addition, instructor attitude also had a positive effect on transforming the use intention of online learning, a result that was consistent with that of a previous study (Rodríguez-Ardura and Meseguer-Artola, 2016). In particular, in a successful online learning system, the teachers' teaching evaluation and real-time response to interaction with students are indispensable elements for the success of online learning (Sun et al., 2008; Al-Rahmi et al., 2019). Teachers adopted online learning to continue teaching during the pandemic, and their help, attention, or advice during online teaching sessions formed motivation for students to switch to online learning. Thus, the effects of pull factors on switching intention strengthened the function and operability of the overall online learning system, improved teacher teaching attitudes, and enable curriculum content to be more in line with the student needs, thereby attracting students to switch to online learning. These aspects constituted the crucial concepts of online learning pull effects.

As anticipated, the habit (mooring factor) of physical learning had a negative impact on switching intention, an observation that is consistent with the results of past studies (Hsieh et al., 2012; Cheng et al., 2019). The results of the present study demonstrated that previous use habits (in physical learning) did not change with user switching intention, indicating that despite the numerous advantages of physical learning over online learning (e.g., more satisfactory interaction, face-to-face communication), students were still reluctant to return to physical courses due to the pandemic. Consequently, despite their previous use habits, students still chose to accelerate their switch to online learning services in a learning environment shaped by emergency management during the COVID-19 pandemic. Finally, habit acted as a moderating effect that interfered with the effects of push and pull factors on switching intention. The results indicated that the influence of habit was non-significant, which differed from previous findings (Jung et al., 2017; Li and Ku, 2018). This suggested that previous use habits did not moderate the push or pull effects on switching intention.

### Implications for Research

This study includes several major contributions. First, from a theoretical perspective, previous switching behavior studies on PPM have tended to discuss long-term-oriented migration behavior, such as switches from Instagram to Facebook (Hou and Shiau, 2020), from E-commerce to M-Shopping or social commerce (Chang et al., 2017; Li and Ku, 2018), or from mobile instant messaging (Sun et al., 2017) or cloud storage services (Wu et al., 2017; Cheng et al., 2019). This study investigated migration behavior during the emergency management implemented by Chinese universities under the impact of the COVID-19 pandemic. Based on the results of this study, different interpretation concepts—for migration behavior under emergency management—are also provided. Second, in the online learning environment, the concept of TTF has not been incorporated into the framework of PPM. This study considered the online environment from the perspective of emergency management of the COVID-19 pandemic, previous studies have emphasized the need to consider the appropriateness of TTF in population migration. Thus, the application and integration of PPM and TTF cover a new perspective and enrich the literature on switching behaviors. Third, this study explored the environment for the switch from offline to online learning. Through evaluation, TTF, instructor attitude, perceived usefulness, and perceived ease of use were regarded as two-level concepts that formed push effects. However, switching behavior-related literature has not investigated the concept of instructor attitude as an influencing push effect and has focused mostly on aspects of student learning (Balakrishnan et al., 2017; Liao et al., 2019). Discussing instructor attitude can further explain the effect of teaching interaction on understanding switching intention in the online teaching environment.

### Limitations and Future Research

This study adopted a questionnaire survey, convenience sampling, and snowball sampling, resulting in a limited scope of samples collected. In particular, the sample attributes did not cover universities in all provinces of China, which can be addressed in subsequent studies. Whether regional characteristics affect the promotion of online learning and student switching intention during the pandemic is also a question worth investigating. Second, the results were still those of a cross-sectional study. For the effects of the continuous development of the COVID-19 pandemic, future researchers are recommended to investigate these through longitudinal studies. Third, the sampling in this study was based on the survey approach. The results were only statistical findings and may not be able to cover more in-depth discussion of the switching intention for online learning promoted during emergency management. Therefore, future studies can focus on qualitative research, which can reveal core factors related to switching intention and produce results that can more effectively explain the quantitative research findings. Fourth, this study did not discuss whether habit could be a moderating effect of the pull/push effects and switching intention. Also, whether sex would be a moderator to the generating of the model, this is also a question could study in the future. Finally, this study explained switching intention for online learning under emergency management using a PPM framework under the impact of the COVID-19 pandemic. Thus, in future discussions of emergency responses in different response environments, this framework can be adopted for an in-depth summary, which can help with understanding factors in different fields. The results can also serve as a reference for various development and service-related applications.

## Data Availability Statement

The original contributions presented in the study are included in the article/supplementary material, further inquiries can be directed to the corresponding author/s.

## Ethics Statement

Ethical review and approval was not required for the study on human participants in accordance with the local legislation and institutional requirements. The patients/participants provided their written informed consent to participate in this study.

## Author Contributions

YJ and C-LL planned the study, collected the data, and wrote the manuscript. QZ, C-LL, and S-WY analyzed the data and wrote the manuscript. YJ, Y-SS, and C-LL was mainly manuscript revised and editing. All authors listed have made a substantial, direct and intellectual contribution to the work, and approved it for publication.

## Conflict of Interest

The authors declare that the research was conducted in the absence of any commercial or financial relationships that could be construed as a potential conflict of interest.
